# *Ganoderma lucidum* promotes sleep through a gut microbiota-dependent and serotonin-involved pathway in mice

**DOI:** 10.1038/s41598-021-92913-6

**Published:** 2021-07-01

**Authors:** Chunyan Yao, Zhiyuan Wang, Huiyong Jiang, Ren Yan, Qianfei Huang, Yin Wang, Hui Xie, Ying Zou, Ying Yu, Longxian Lv

**Affiliations:** 1 Key Laboratory of Nutrition of Zhejiang Province, Institute of Health Food, Hangzhou Medical College, Hangzhou, Zhejiang China; 2 Animal Center, Hangzhou Medical College, Hangzhou, Zhejiang China; 3 State Key Laboratory for Diagnosis and Treatment of Infectious Diseases, Collaborative Innovation Center for Diagnosis and Treatment of Infectious Diseases, The First Affiliated Hospital, College of Medicine, Zhejiang University, Hangzhou, Zhejiang China; 4 The Second Affiliated Hospital of Zhejiang, Chinese Medical University, Hangzhou, Zhejiang China

**Keywords:** Neuroscience, Circadian rhythms and sleep

## Abstract

*Ganoderma lucidum* is a medicinal mushroom used in traditional Chinese medicine with putative tranquilizing effects. However, the component of *G. lucidum* that promotes sleep has not been clearly identified. Here, the effect and mechanism of the acidic part of the alcohol extract of *G. lucidum* mycelia (GLAA) on sleep were studied in mice. Administration of 25, 50 and 100 mg/kg GLAA for 28 days promoted sleep in pentobarbital-treated mice by shortening sleep latency and prolonging sleeping time. GLAA administration increased the levels of the sleep-promoting neurotransmitter 5-hydroxytryptamine and the *Tph2, Iptr3* and *Gng13* transcripts in the sleep-regulating serotonergic synapse pathway in the hypothalamus during this process. Moreover, GLAA administration reduced lipopolysaccharide and raised peptidoglycan levels in serum. GLAA-enriched gut bacteria and metabolites, including *Bifidobacterium*, *Bifidobacterium animalis*, indole-3-carboxylic acid and acetylphosphate were negatively correlated with sleep latency and positively correlated with sleeping time and the hypothalamus 5-hydroxytryptamine concentration. Both the GLAA sleep promotion effect and the altered faecal metabolites correlated with sleep behaviours disappeared after gut microbiota depletion with antibiotics. Our results showed that GLAA promotes sleep through a gut microbiota-dependent and serotonin-associated pathway in mice.

## Introduction

Sleep is important for all living species, and it comprises roughly one-third of our lives. Good sleep is a prerequisite for physical function (including promoting growth, learning and cognitive development), immunity^1^, and poor or insufficient sleep is currently an important health problem^2^. Currently, 15–35% of adults suffer from regular sleep disruptions, such as difficulties in initiating sleep, insufficient sleep time or frequent waking during the night^3^. Sleep problems may damage daytime lives by feeling exhausted and making troubles^4^. Moreover, persistent sleep problems are frequently associated with cardiovascular diseases^5^, obesity, diabetes^6,7^, and mortality^8^.

*Ganoderma lucidum*, also known as Lingzhi in China, is a medicinal mushroom that has been widely used for medicinal purposes over the past 2000 years^9^, including for tranquilizing. The tranquilizing effect of *G. lucidum's* was recorded in “Shennong's herbal classic” as early as the first century BC and currently in the Chinese Pharmacopoeia^10^. Recently, it was reported that *G. lucidum* improved the sleep of patients with insomnia or other mental disorders^11,12^. Furthermore, *G. lucidum* promoted sleep in pentobarbital-treated mice and rats^13^. However, most sleep-related experiments are based on the water extract of *G. lucidum*. The effect of other extract parts on sleep has not been explored to date.

Recent studies indicate that alterations in the gut microbiota might be associated with sleep through the gut–brain axis^14,15^. Structural components of the microbial cell wall, such as lipopolysaccharide (LPS) and peptidoglycan (PG), stimulate the innate immune system that begins at the intestinal mucosal surface and impacts the entire body^14^. Anderson et al. found that better sleep quality was associated with higher proportions of the gut microbial phyla *Verrucomicrobia* and *Lentisphaerae* in healthy adults, suggesting a possible relationship between sleep quality and the gut microbiota^15^. Poroyko et al. showed that chronic sleep fragmentation (SF) was related to the gut microbiota through conventionalization of germ-free mice with the gut microbiota of mice with SF^16^.

In this work, the effect of the acidic part of the alcohol extract of *G. lucidum* mycelia (GLAA) on sleep was investigated using a hypnotized mouse model induced by pentobarbital sodium. Furthermore, the underlying mechanism was explored by integrating microbiomics, transcriptomics, and metabolomics and then verified using gnotobiotic mice.

## Results

### GLAA has no obvious toxicological effect on mice

**Figure 1:**
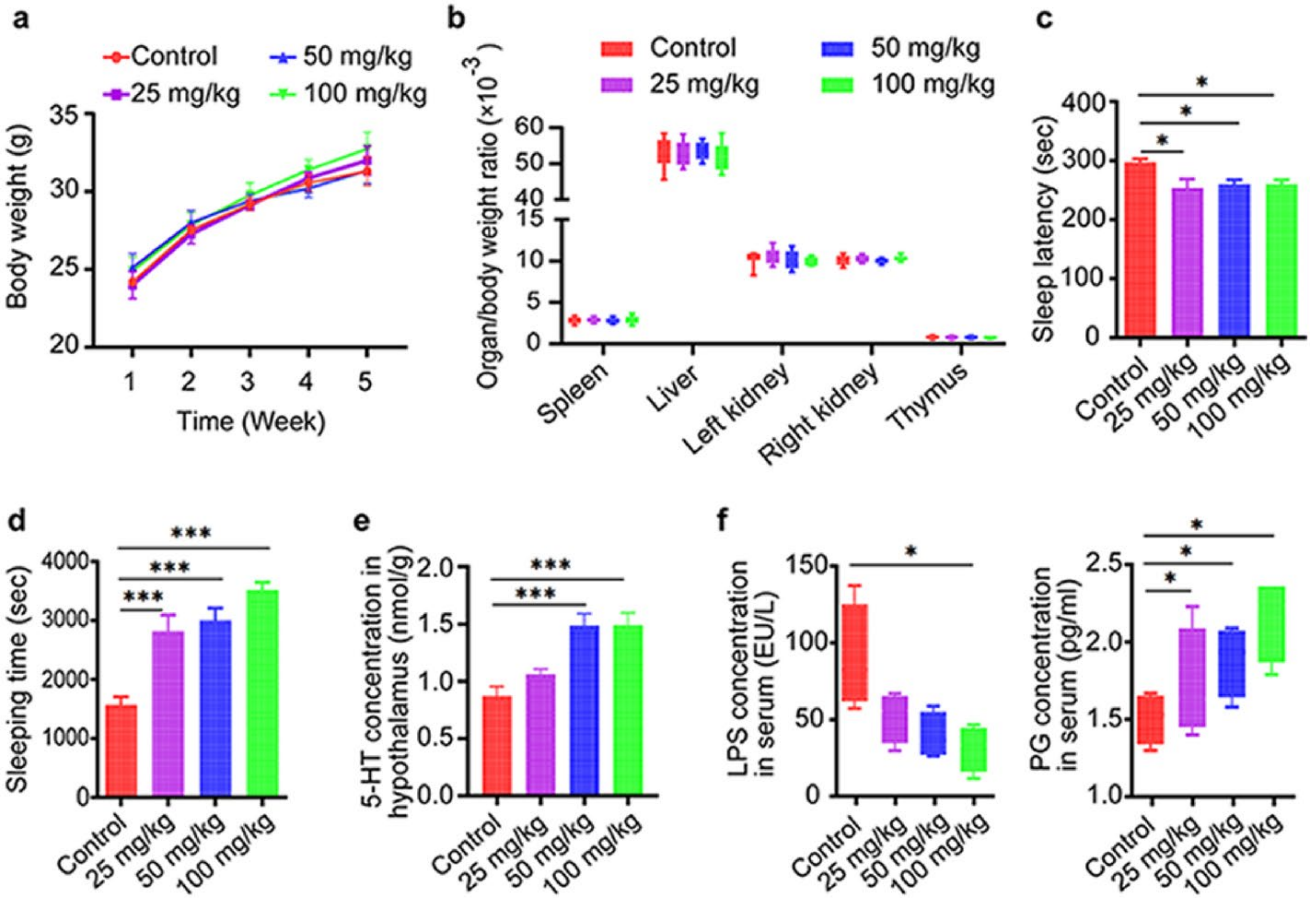
Gavage administration of GLAA promoted sleep in mice with pentobarbital-induced hypnosis. (**a**) GLAA did not affect the weight of mice. (**b**) GLAA did not alter the organ/body weight ratio of the spleen, liver, kidney or thymus. (**c**) GLAA promoted sleep by shortening sleep latency. (**d**) GLAA prolonged sleeping time. (**e**) GLAA increased the concentration of 5-HT in the hypothalamus (n = 4–5). (**f**) GLAA reduced the LPS content and raised the PG content in serum. The data are shown as the means ± SEMs; *p < 0.05, **p < 0.01, ***p < 0.001, and ‘ns’ indicates that the difference is not significant. The data are calculated from 10 samples per group, except specifically mentioned. And all the images are generated by GraphPad Prism 8. The animal experiment and the subsequent behavioural evaluation were performed twice. *GLAA* The acidic part of the alcohol extract of *G. lucidum* mycelia, *5-HT* 5-hydroxytryptamine, *LPS* lipopolysaccharide, *PG* peptidoglycan.

Body weight was evaluated every week, and administration of GLAA did not affect mouse weight (Fig. 1a). After the mice were sacrificed, the spleen, liver, kidney and thymus were observed by a pathologist who was blind to our experiment, and no obvious pathological changes were found in these organs. Moreover, pre-treatment with GLAA also did not alter the organ/body weight ratio of these organs (Fig. 1b). In addition, administration of GLAA did not induce histological abnormalities in the colons of mice (Supplementary Fig. S1). These results showed that GLAA was safe for mice at dose from 25 to 100 mg/kg.

### GLAA potentiates pentobarbital-induced sleep

Using a mouse model of pentobarbital-induced hypnosis, we observed that gavage administration of GLAA for 4 weeks led to significant decreases in sleep latency and increases in sleeping time compared with the solvent control containing 0.05% sodium carboxymethyl acid (0.05% CMC-Na). The average sleep latency of the control group was approximately 300 s, while those of groups administered GLAA at levels ranging from 25 to 100 mg/kg were shortened to approximately 250 s (Fig. 1c). The sleeping time of mice gavaged with 25 mg/kg, 50 mg/kg and 100 mg/kg GLAA increased significantly from 1570.4 s (at 0 mg/kg) to 2816.5 s, 3000 s and 3523.5 s, respectively, showing a certain dose dependency (Fig. 1d). These results suggested that gavage administration of GLAA could significantly promote the sleep quality of mice.

### GLAA increases the 5-HT content in the hypothalamus

To determine which neurotransmitters were responsible for the sleep changes in mice, 5-HT, GABA, norepinephrine and dopamine were detected in the hypothalamus of mice with a commercial ELISA kit. Only the 5-HT level was significantly increased after administration of 50 mg/kg and 100 mg/kg GLAA (Fig. 1e), indicating that 5-HT may act as a target neurotransmitter for GLAA to promote sleep health in mice.

### GLAA reduces the LPS content and increases the PG content in serum

LPS and PG are two major components of the bacterial cell wall and two indicators of body inflammation^14^. LPS exists in the cell wall of only gram-negative bacteria, and the PG content in the cell wall of gram-positive bacteria is much higher than that in the cell wall of gram-negative bacteria^17,18^. In our study, administration of GLAA significantly reduced the LPS content but raised the PG content in serum (Fig. 1f), indicating that GLAA may change the composition of the gut microbiota.

### GLAA changes the composition of the gut microbiota

**Figure 2:**
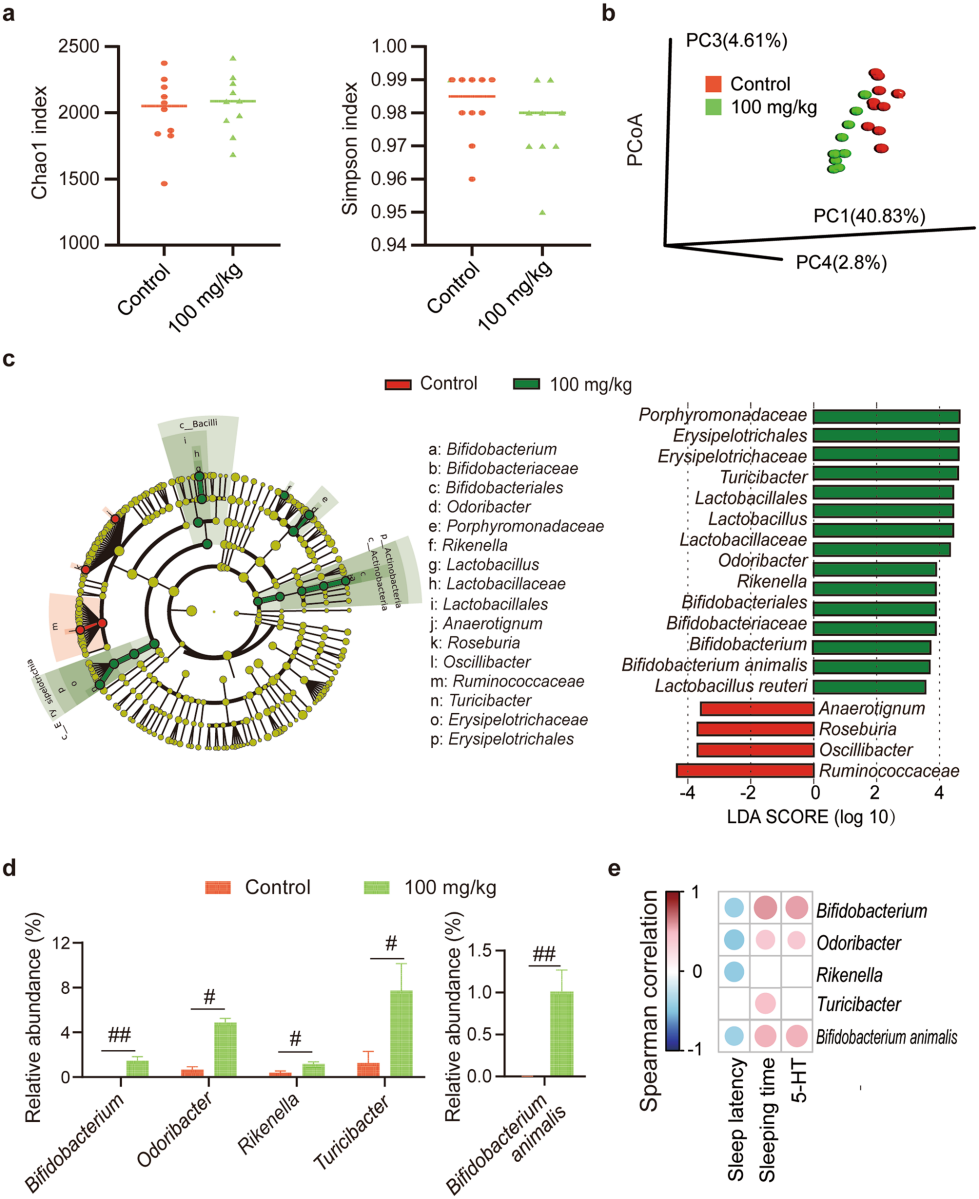
Gavage administration of GLAA induced alterations in the gut microbiota that were correlated with sleep behaviour in mice. (**a**) The richness and diversity of the faecal microbiota were evaluated by the Chao1 index and the Simpson index respectively. Values are expressed as the median with interquartile range. (**b**) Principal coordinate analysis (PCoA) plot with unweighted UniFrac distances based on operational taxonomic units (OTUs) of the gut microbiota. The image is generated by R package “ade4”. (**c**) The relative abundance of gut bacterial taxa differed between the control group and the 100 mg/kg group and was calculated by LEfSe analysis (LDA score > 3.5). The image is generated on line at the Huttenhower Lab (http://huttenhower.sph.harvard.edu/galaxy). The taxa enriched in the control group are shown in red, and the taxa in the 100 mg/kg group are shown in green. (**d**) The discriminative bacteria altered by 100 mg/kg GLAA (LDA score > 3.5 and *p*_adj_ < 0.05). (**e**) Correlations of the discriminative bacteria, sleep behaviour and the concentration of 5-HT in the hypothalamus were determined using Spearman’s rank correlation analysis. The image is generated by R package “corrplot”. The results with p values < 0.05 are shown in the heat map. The colour key and circle size indicate the strength of the correlation (r value). Red indicates positive correlations; blue indicates negative correlations. All the data were calculated from 10 samples per group. The data are shown as the means ± SEMs; ^#^*p*_adj_ < 0.05, ^##^*p*_adj_ < 0.01. Except specifically mentioned, the images are generated by GraphPad Prism 8. *G*^*+*^ Gram-positive, *GLAA* the acidic part of the alcohol extract of *G. lucidum* mycelia, *5-HT* 5-hydroxytryptamine.

Next-generation sequencing of 16S rRNA V3–V4 regions was conducted to investigate the faecal microbiota composition. As shown by the Chao1 index, the microbiota of the control and 100 mg/kg GLAA groups exhibited the same richness (Fig. 2a); as shown by the Simpson index, there was also no significant difference in microbiota diversity between the control group and the 100 mg/kg GLAA group (Fig. 2a). However, the principal coordinate analysis (PCoA) results showed that the microbiota profiles of the two groups were clearly separated from each other (Fig. 2b). PERMANOVA further confirmed that the compositions were different between the control and 100 mg/kg GLAA groups (p = 0.001).

To analyse the effect of 100 mg/kg GLAA on the intestinal microbiota, discriminative taxa were identified by LEfSe analysis at multiple levels, and the most different results (linear discriminant analysis (LDA) score > 3.5) were retained to investigate the alteration in the microbiota structure (Fig. 2c). Compared with the control group, the 100 mg/kg group exhibited enrichment of the family *Bifidobacteriaceae* and its affiliated genus *Bifidobacterium* and species *Bifidobacterium animalis*, the family *Lactobacillaceae* and its affiliated genus *Lactobacillus* and species *Lactobacillus reuteri*, the family *Porphyromonadaceae* and its affiliated genus *Odoribacter*, the family *Erysipelotrichales* and its affiliated genus *Turicibacter*, and the genus *Rikenella*. In addition, administration of 100 mg/kg GLAA depleted the family *Ruminococcaceae* and its affiliated genus *Oscillibacter* and the genera *Anaerotignum* and *Roseburia*. Finally, 100 mg/kg GLAA significantly enriched *Bifidobacterium*, *Rikenella*, *Odoribacter* and *Turicibacter* at the genus level, as well as *Bifidobacterium animalis* at the species level after p value correction (*p*_adj_ < 0.05, Fig. 2d).

The correlation of discriminative bacterial genera with the sleep behavioural index and concentration of 5-HT in the hypothalamus were determined using Spearman’s rank correlation analysis (Fig. 2e). *Bifidobacterium*, *Odoribacter*, *Rikenella* and *Turicibacter* at the genus level, as well as *Bifidobacterium animalis* at the species level, were negatively correlated with sleep latency. *Bifidobacterium* and *Odoribacter* at the genus level, as well as *Bifidobacterium animalis* at the species level, were positively correlated with sleeping time and the concentration of 5-HT in the hypothalamus. These results indicated that the gut microbiota may be involved in the process by which GLAA promotes sleep in mice.

### GLAA alters gut metabolites

**Figure 3:**
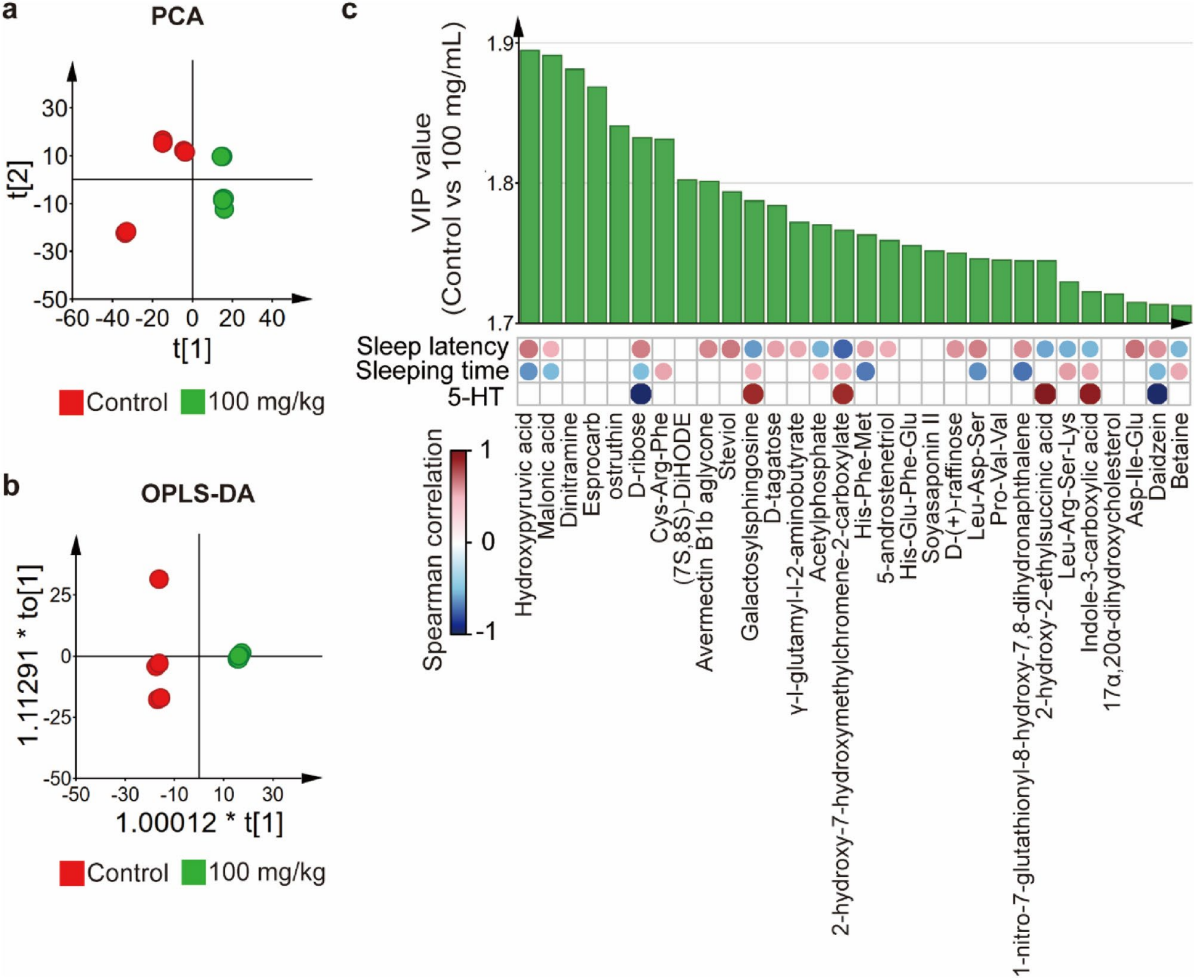
Gavage administration of GLAA led to alterations in gut metabolites that were correlated with sleep behaviour in mice. (**a**) PCA score plots of metabolome profiles of the control group and the 100 mg/kg GLAA group. (**b**) OPLS-DA score plots of metabolome profiles of the control group and the 100 mg/kg GLAA group. (**c**) Correlations of the top 30 VIP (variable importance in projection) gut metabolites, sleep behaviour and the concentration of 5-HT in the hypothalamus were determined using Spearman’s rank correlation analysis. The image is generated by SIMCA 14.1 and R package “corrplot”. The results with p values < 0.05 are shown in the heat map. The colour key and circle size indicate the strength of the correlation (r value). Red indicates positive correlations; blue indicates negative correlations. The data are calculated from 8 samples per group. Except specifically mentioned, the images are generated by SIMCA 14.1. *GLAA* the acidic part of the alcohol extract of *G. lucidum* mycelia, *PCA* principal component analysis, *OPLS-DA* orthogonal partial least squares discriminant analysis, *VIP* variable importance in projection, *5-HT* 5-hydroxytryptamine.

To explore the relationship between alterations in gut metabolites and sleep, faecal samples were submitted to metabolome analysis using LC–MS/MS. In total, 13,023 metabolites were identified. 1037 metabolites with score > 0.5 and a level 2 identification were retained (supplemental material 1)^19^. Principal component analysis (PCA) showed that the control group and 100 mg/kg group clustered separately from each other in both the original identification metabolome profiles and the retained metabolome profiles, suggesting that the metabolome profiles of these two groups (Fig. 3a, Supplementary Fig. S2a) are quite different. Similar results were obtained from the orthogonal partial least squares discriminant analysis (OPLS-DA) (Fig. 3b, Supplementary Fig. S2b).

The top 30 metabolites were selected based on the variable importance in the projection (VIP) value from the retained metabolome profiles to explain the difference between the two groups in the OPLS-DA model (Fig. 3c), which most contributed to the alteration of the metabolome between the control group and the 100 mg/kg group. In addition, all the top 30-VIP metabolites showed significant difference (*p*_adj_ < 0.05). After gavage administration of 100 mg/kg GLAA, hydroxypyruvic acid, malonic acid, dinitramine, esprocarb, ostruthin, d-ribose, (7S,8S)-DiHODE, avermectin B1b aglycone, steviol, d-tagatose, γ-l-glutamyl-l-2-aminobutyrate, His-Phe-Met, 5-androstenetriol, His-Glu-Phe-Glu, soyasaponin II, d-(+)-raffinose, Leu-Asp-Ser, 1-Nitro-7-glutathionyl-8-hydroxy-7,8-dihydronaphthalene, Asp-Ile-Glu and daidzein were enriched in faeces, while Cys-Arg-Phe, galactosylsphingosine, acetylphosphate, 2-hydroxy-7-hydroxymethylchromene-2-carboxylate, Pro-Val-Val, 2-hydroxy-2-ethylsuccinic acid, Leu-Arg-Ser-Lys, indole-3-carboxylic acid, 17α,20α-dihydroxycholesterol and betaine were depleted in the gut.

Spearman’s rank correlation analysis showed that most metabolites were correlated with the sleep behavioural indexes and concentration of 5-HT in the hypothalamus (Fig. 3c). d-ribose and daidzein showed negative correlation with sleep-promotion index and hypothalamic 5-HT concentration. On the contrary, galactosylsphingosine, 2-hydroxy-7-hydroxymethylchromene-2-carboxylate, 2-hydroxy-2-ethylsuccinic acid and indole-3-carboxylic acid were positively correlated with sleep-promotion index and hypothalamic 5-HT concentration. Hydroxypyruvic acid, malonic acid, avermectin B1b aglycone, steviol, d-tagatose, γ-l-glutamyl-l-2-aminobutyrate, His-Phe-Met, 5-androstenetriol, d-(+)-raffinose, Leu-Asp-Ser, 1-Nitro-7-glutathionyl-8-hydroxy-7,8-dihydronaphthalene and Leu-Arg-Ser-Lys were correlated with at least one sleep behavioural index, which suggested that they were positively correlated with sleep-promotion effect. Conversely, Cys-Arg-Phe, acetylphosphate, Leu-Arg-Ser-Lys and betaine exhibited negatively correlated with sleep-promotion effect.

### Antibiotic-induced depletion of the gut microbiota inhibits the sleep-promoting effect and the increase in 5-HT caused by GLAA

**Figure 4:**
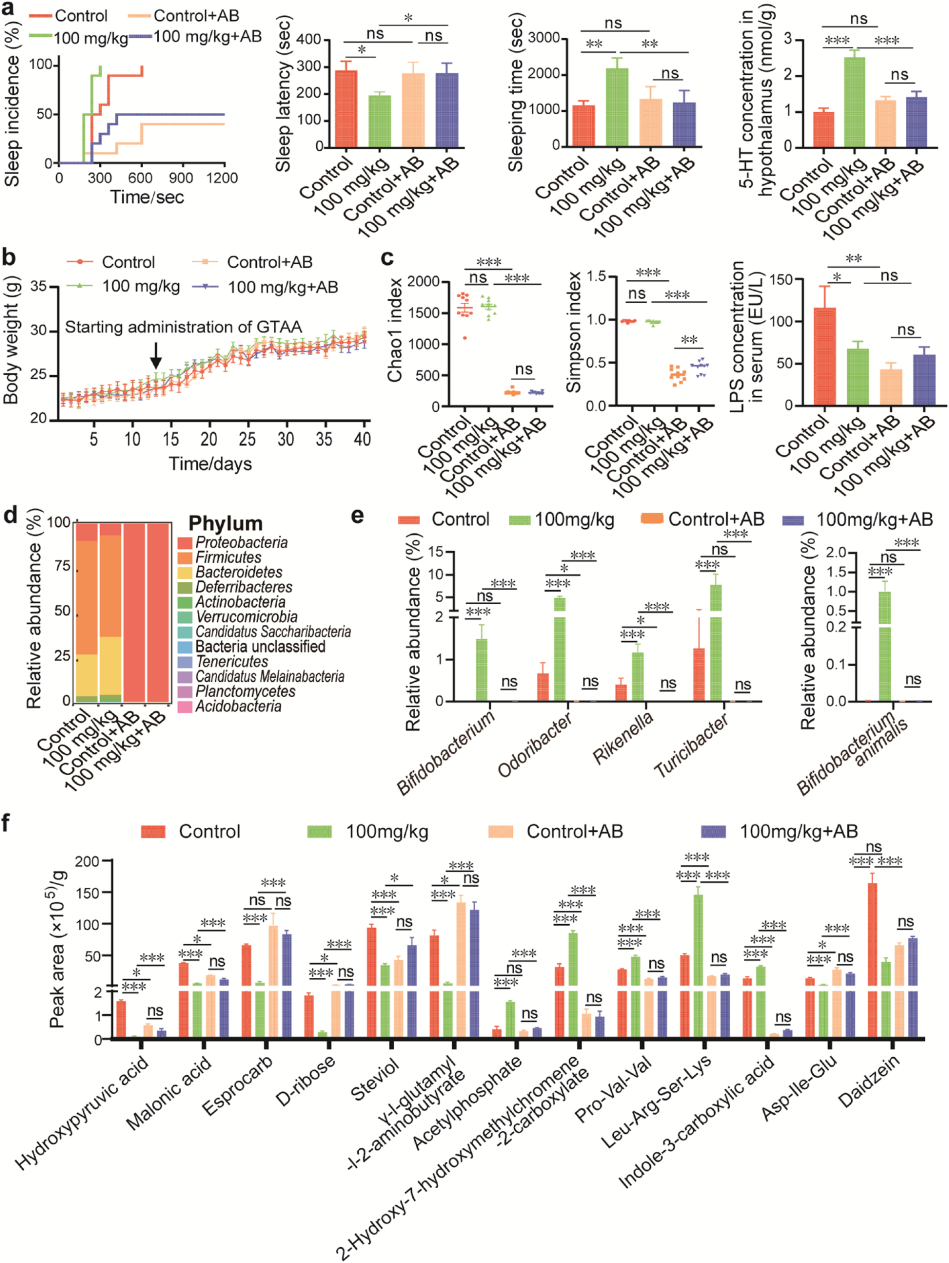
The sleep promotion effects of GLAA were dependent on the gut microbiota. (**a**) Antibiotics blocked GLAA-induced alterations in sleep behaviour and increases in 5-HT (n = 4–5). (**b**) Antibiotic treatment did not affect the body weight of mice. (**c**) Antibiotic treatment decreased microbiota richness, diversity and serum gram-negative bacterial component levels, as evaluated by the Chao1 index, Simpson index and serum LPS content. (**d**) Composition of the faecal microbiota after antibiotics treatment at the phylum level. The image is generated by R package “ggplot2”. (**e**) Effects of GLAA on the gut microbiota of mice treated with antibiotics. (**f**) Antibiotics partially depleted gut metabolites altered by GLAA. The data are shown as the means ± SEMs; *p < 0.05, **p < 0.01, ***p < 0.001, ‘ns’ indicates that the difference is not significant. All the data are calculated from 8 to 10 samples per group, and the images are generated by GraphPad Prism 8, except specifically mentioned. *GLAA* the acidic part of the alcohol extract of *G. lucidum* mycelia, *AB* antibiotics, *5-HT* 5-hydroxytryptamine, *LPS* lipopolysaccharide.

To explore whether the sleep promotion effect of GLAA was dependent on the gut microbiota, we depleted the gut microbiota in mice using antibiotics according to a previous report^20^. After 12 days of depletion of the gut microbiota using an antibiotic concoction, GLAA was gavaged from the 13th day to the 40th day, together with the antibiotic concoction. Notably, neither antibiotic treatment nor GLAA gavage had a significant effect on the body weight of mice (Fig. 4b).

Then, a mouse model of the pentobarbital hypnosis test was established. All the control mice fell asleep within 600 s, while all mice administered 100 mg/kg GLAA fell asleep within 300 s. However, mice pre-treated with antibiotic concoction exhibited only 40% sleep incidence during the experimental period. Even after administration of 100 mg/kg GLAA, the incidence of sleep in mice pre-treated with antibiotic concoction was only 50% (Fig. 4a). Furthermore, pre-treatment with antibiotic concoction inhibited the increase in 5-HT induced by 100 mg/kg GLAA, as observed in mice (Fig. 4a). Therefore, depletion of the gut microbiota prevented the sleep promotion effect of GLAA, indicating that gut microbiota might play a pivotal role in GLAA-mediated promotion of sleep.

Our results showed that antibiotic treatment depleted more than 85% of the operational taxonomic units (OTUs) of gut bacteria, with a significant decrease in the Chao1 and Simpson indexes (Fig. 4c); moreover, the significant decrease in the LPS content in serum further indicated a decrease in microbial quantity in the gut (Fig. 4c). The remaining bacteria mainly included *Proteobacteria* (99.35%) at the phylum level (Fig. 4d), *Enterobacteriaceae* (99.06%) at the family level (Supplementary Fig. S3a), and *Klebsiella* (81.4%) and *Proteus* (13.8%) at the genus level (Supplementary Fig. S3b). In contrast, in the control and 100 mg/kg groups without antibiotics, the microbiota presented a normal distribution of *Firmicutes* (63.6% and 56.96%), *Bacteroidetes* (23.5% and 32.38%), *Proteobacteria* (9.6% and 6.47%), *Deferribacteres* (2.9% and 2.3%), and *Actinobacteria* (0.1% and 1.64%), with other phyla accounting for less than 0.2% (Fig. 4d). The above-mentioned microbial markers altered by GLAA were all depleted after antibiotic treatment, and this depletion was not reversed by GLAA in the antibiotic-gavaged mice (Fig. 4e).

Among the top 30 faecal metabolites altered by 100 mg/kg GLAA, the alterations in 13 metabolites were totally or partially prevented by depletion of the gut microbiota (Fig. 4f). Depletion of the gut microbiota prevented the GLAA-induced elevation of acetylphosphate, 2-hydroxy-7-hydroxymethylchromene-2-carboxylate, Pro-Val-Val, Leu-Arg-Ser-Lys and indole-3-carboxylic acid. It also prevented the GLAA-induced depletion of hydroxypyruvic acid, Asp-Ile-Glu and daidzein. Notably, except for esprocarb, the remaining 12 of the above-mentioned 13 metabolites were strongly correlated with sleep latency, sleeping time and/or the concentration of 5-HT in the hypothalamus.

### GLAA regulates the serotonergic synapse pathway

**Figure 5:**
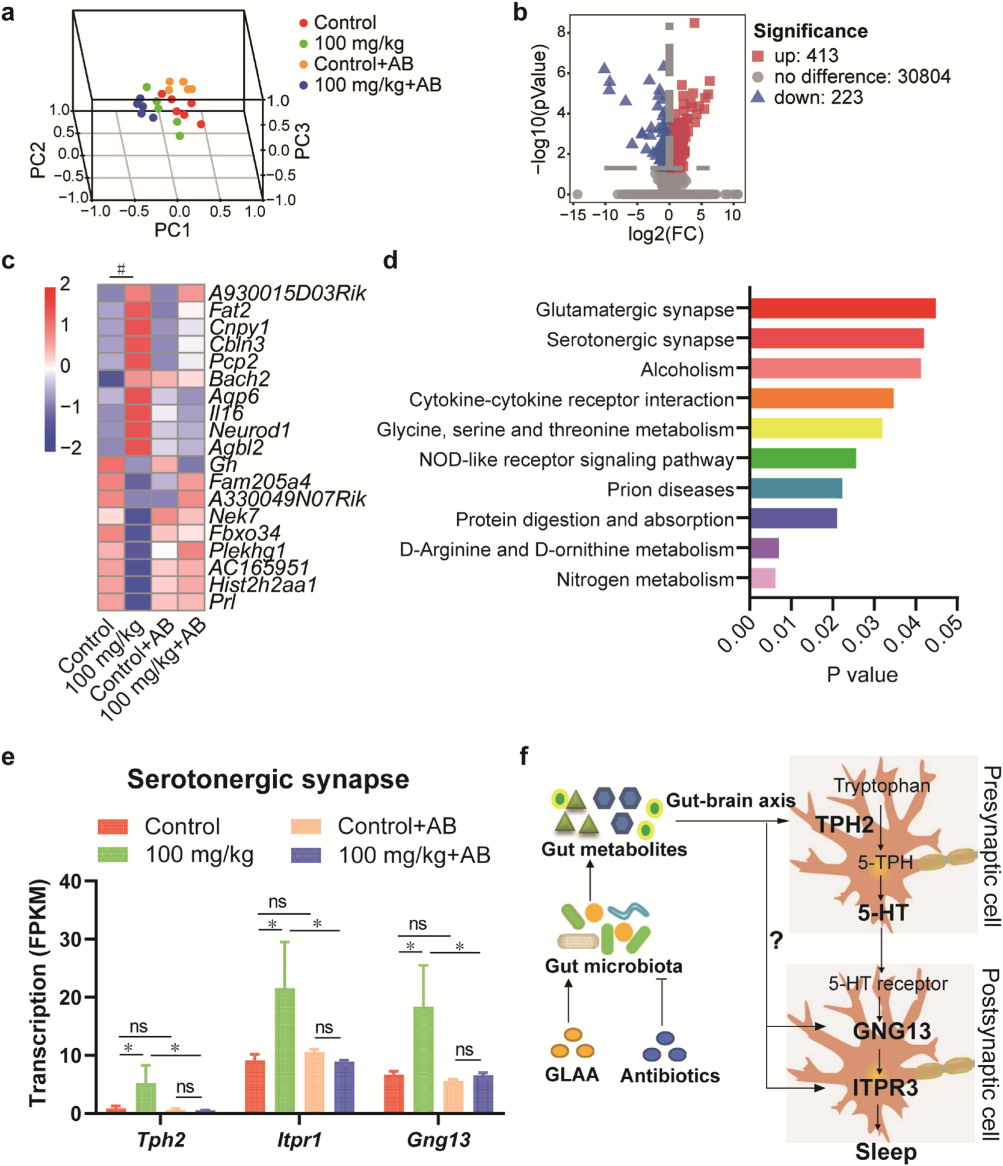
Gavage administration of GLAA regulated the serotonergic synapse pathway in the hypothalamus. (**a**) PCA score plots of transcriptome profiles of these four groups. (**b**) Volcano map of gene transcription after administration of GLAA. (**c**) Gene transcription was altered after GLAA administration in the four groups (*p*_*adj*_ > 0.05). (**d**) KEGG pathways altered in the hypothalamus after administration of 100 mg/kg GLAA (*p* > 0.05). (**e**) Gene transcription in the serotonergic synapse pathway was altered after GLAA administration in the four groups. (**f**) General view of the effects of GLAA on sleep promotion. The data are calculated from 5–6 samples per group. The data are shown as the means ± SEMs; *p < 0.05, ^#^*p*_adj_ < 0.05, ‘ns’ indicates that the difference is not significant. The images a, b and c are generated by R package “scatterplot3d”, “ggplot2” and “pheatmap” respectively, the image f is generated by ScienceSlides Suite, and the others are generated by GraphPad Prism 8. *GLAA* the acidic part of the alcohol extract of *G. lucidum* mycelia, *AB* antibiotics, *PCA* principal component analysis, *5-HT* 5-hydroxytryptamine.

Genome-wide transcriptional profiling was performed to analyse the changes in gene transcription in the hypothalamus of mice. In total, 830,782,772 valid sequencing reads were generated from 21 samples (5–6 samples per group), and 55,450 expressed genes were identified after mapping to the mouse genome. The PCA results showed that the transcriptional profiles were clearly separated from each other (Fig. 5a).

After administration of 100 mg/kg GLAA for 4 weeks, the transcripts of 413 genes were upregulated, and the transcripts of 223 genes were downregulated (p < 0.05 and |log2(FC)|≥ 1) in the hypothalamus compared with the levels in control mice (Fig. 5b). Among these genes, 20 genes with known structure and/or function were obtained after p value correction (*p*_adj_ < 0.05, Fig. 5c). Pre-treatment with 100 mg/kg GLAA upregulated *A930015D03Rik*, *Fat2*, *Cnpy1*, *Cbln3*, *Pcp2*, *Bach2*, *Aqp6*, *Il16*, *Neurod1* and *Agbl2*. These genes were mainly related to the structural (*Fat2*, *Cnpy1*, *Cbln3*, *Aqp6* and *Neurod1*) and immune (*Pcp2*, *Il16* and *Agbl2*) development of the brain. Some of these genes also have antitumour and anti-inflammatory effects, such as *Bach2* and *Agbl2*. In contrast, gavage administration of 100 mg/kg GLAA downregulated *Gh*, *Fam205a4*, *A330049N07Rik*, *Nek7*, *Fbxo34*, *Plekhg1*, *AC165951*, *Hist2*, *h2aa1* and *Prl* expression. Importantly, *Gh* (growth hormone) and *Prl* (prolactin) are two hormone-encoding genes, and low expression of these genes was reported to be associated with decreasing wakefulness and increasing slow-wave sleep^21,22^.

All the significant genes mapped by KEGG Mapper revealed that administration of 100 mg/kg GLAA regulated 10 pathways, namely, nitrogen metabolism; d-arginine and d-ornithine metabolism; protein digestion and absorption; prion diseases; NOD-like receptor signalling pathway; glycine, serine and threonine metabolism; cytokine–cytokine receptor interaction; alcoholism; glutamatergic synapse; and serotonergic synapse (Fig. 5d). These pathways were mostly involved in sleep (glutamatergic synapse and serotonergic synapse) and immunity (prion diseases, NOD-like receptor signalling pathway and cytokine–cytokine receptor interaction) regulation^23,24^.

Then, we specifically examined the serotonergic synapse, the output of which is 5-HT. After gavage administration of 100 mg/kg GLAA, the transcription levels of *Tph2*, *Itpr2* and *Gng13* in the serotonergic synapse pathway increased significantly, and depletion of the gut microbiota also blocked these increases. Tryptophan is converted by the enzyme tryptophan hydroxylase (TPH) to 5-hydroxytryptophan (5-HTP), and 5-HTP is further converted to 5-HT in presynaptic cells^25^. 5-HT is secreted into postsynaptic cells and binds to 5-HT receptors (mainly 5-HT1R, 5-HT2R and 5-HT5R) based on the KEGG pathway, thus activating downstream signalling pathway-related genes (including *Itpr2* and *Gng13*) and promoting sleep (Fig. 5e,f).

## Discussion

Sleep problems have become a hot topic of global concern*. G. lucidum* has been used as a tranquilizing agent for the treatment of restlessness, insomnia, and palpitation in China for hundreds of years^26^. Chu et al. reported that 80 mg/kg and 120 mg/kg water extracts of *G. lucidum* prolonged the sleeping time of mice by 50% and 60%, respectively, while a 40 mg/kg dose did not significantly increased the sleeping time^13^. In this study, for the first time, the acidic part of the ethanol extract of *G. lucidum* was proven to promote sleeping time by 79.35% (25 mg/kg), 91.03% (50 mg/kg) and 124.37% (100 mg/kg). The sleep promotion effect of GLAA in this study seemed to be better than that of the water extract of *G. lucidum* according to published data, although this conclusion was limited by comparisons between different studies.

The ethanol extract of *G. lucidum* was reported to contain approximately 130–140 triterpene acids, 20 sterols, fewer alkaloids and other compounds^27,28^. The preparation of GLAA used in our study is also the usual method to enrich triterpene acids that are one of the main medicinal ingredients of the ethanol extract of *G. lucidum*, playing important roles in the anti-inflammatory, antioxidant and antitumour activities of this mushroom^29–31^. The compositions of GLAA are also very complicated. It was reported that the main component of this part was triterpene acids, although the normal content of triterpene acids in different sources of *G. lucidum* ranges from 6 to 16%^29^. However, it is not known whether the sleep promoting effect of GLAA is mediated by triterpene acids.

Various neurotransmitters, including GABA, 5-HT, norepinephrine and dopamine, affect different brain nuclei to regulate the switch between wakefulness and sleep^32^. In this study, GLAA was found to promote sleep through a 5-HT associated pathway. The monoamine neurotransmitter 5-HT, also known as serotonin, is important for sleep regulation, and animals deficient in 5-HT synthesis have been shown to stay awake longer and sleep less^32^. Moreover, several researchers have found that increasing the 5-HT content in the brain would prolongs sleeping time in animals, which is consistent with our results. In addition to neurotransmitters, other factors could also affect sleep, such as changes in brain structure, ion channels^33^ and growth hormone^21,22^, some of which were also found in our study.

Pentobarbital is a well-known sedative‐hypnotic and anaesthetic whose hypnotic effect can be enhanced by calcium channel blockers, such as diltiazem and some plant extracts^34^. Ca^2+^ influx and Ca^2+^ channels are widely involved in sleep/wake regulation^35^. Consequently, sleep latency and sleeping time in the pentobarbital-induced sleep test are commonly used as indicators for assessing the sedative and hypnotic effects of dietary supplements (most of them are plant extracts) and drugs such as diazepam (a longer-acting benzodiazepine) and 5-HTP^32^. Several studies have reported that serotonergic synapse played negative roles in calcium currents/channels^35^. Christopher et al. found that serotonin inhibited calcium transients in mesopontine cholinergic neurons of the laterodorsal tegmental nucleus^36^. Douglas et al. showed that serotonin inhibited N- and P/Q-type calcium channels in caudal raphe neurons of neonatal rat brain slices^37^. Therefore, the synergistic effect of GLAA and pentobarbital in the present study may be related to 5-HT-mediated calcium blockade.

Many studies have shown that sleep under normal physiological conditions may be influenced by the gut microbiota^38^. PG and LPS are two bacterial cell wall components that are enriched in gram-positive bacteria and gram-negative bacteria, respectively^38^. Our results showed that both PG and LPS levels were altered in serum after gavage administration of GLAA, indicating that GLAA-induced sleep may be related to the gut microbiota. In addition, GLAA may also affect serum LPS/PG levels by regulating gut barrier integrity and preventing translocation into the circulation according to the results of other *G. lucidum* specimens^39^. Using a mouse model in which the gut microbiota was depleted with antibiotics, we further confirmed that the gut microbiota was necessary for GLAA-mediated promotion of sleep in mice. Interestingly, *Bifidobacterium* and *Odoribacter*, which were enriched in the GLAA group, were highly correlated with sleep behaviour and 5-HT in our study. *Bifidobacterium longum* was reported to upregulate serotonin transporter expression in intestinal epithelial cells in vitro^40^. Bubie et al. found that variation in *Odoribacter* abundance was the likely regulator of sleep architecture, and *Odoribacter* abundance was correlated with sleep phenotypes in mice^41^. Therefore, these results provide a promising prospect for the study of the role and mechanism of intestinal microorganisms in the promotion of sleep.

Sleep restriction and irregular sleep patterns have been associated with metabolic impairments^42^. Our results showed that more than half of faecal metabolites were correlated with sleep latency, sleeping time and 5-HT. Some of them are associated with sleep. d-ribose was found to improve in energy, sleep, mental clarity, pain intensity and well-being of Fibromyalgia and chronic fatigue syndrome^43^. Steviol could increase the uptake of myocardial and cerebral glucose that play roles in sleep behaviour disorder^44,45^. Indole-3-carboxylic acid is synthesized from tryptophan that can also convert into 5-hydroxytryptophan, the precursor of 5-HT^46^. Acetyl-phosphate can induce Ca^2+^–Ca^2+^ exchange in sarcoplasmic reticulum vesicles^47^. Some metabolites are related with function of brain and neurological diseases. Daidzein can affect various neurobiological regulatory mechanisms such as behaviour, cognition, growth, development and reproduction^48^. Betaine (*n*-trimethylglycine), a common osmolyte, has function in the modulation of hippocampal neurophysiology and neuroprotection^49^. Interestingly, two herbicides dinitramine and esprocarb were found in these metabolites, although they were not correlated with sleep behaviour or indicators. And we suspected that they might come from the bedding material. Additionally, due to the nontargeted metabolome analysis with LC–MS/MS, integrated database and more included compounds in the database of our study, a large number of metabolites have been explored, which provides us with more ideas about sleep, but these metabolites and results need further verification and research.

Recently, bidirectional interactions between the central nervous system and the gastrointestinal tract have attracted increasing attention. Preclinical evidence supports a role of the gut microbiota in behavioural responses associated with pain, emotion, autism, anxiety, depression, and sleep^50^. The microbiota and the brain communicate with each other via various routes, including the immune system, endocrine and neurocrine pathways, the vagus nerve and the enteric nervous system, involving microbial metabolites such as short-chain fatty acids, branched-chain amino acids, and peptidoglycans^51^. According to our results, 5-HT and the serotonergic synapse pathway might be among the routes connecting the gut microbiota and sleep signals in the brain.

In summary, in our study, the effect of the acidic part of the alcohol extract of *G. lucidum* mycelia on sleep was studied to show that GLAA significantly shortened sleep latency and prolonged sleeping time in pentobarbital-treated mice. This effect was mainly related to an increase in serotonergic synapse signalling in the hypothalamus and was dependent on the gut microbiota of mice.

## Materials and methods

### Preparation of GLAA

*Ganoderma lucidum* CGMCC5.616 was provided by Zhejiang Wuyangtang Pharmaceutical Incorporated Company, Ltd. GLAA was prepared as described with minor modifications^52^. Briefly, 100 g of the *G. lucidum* fruiting body was crushed, passed through a 20-mesh sieve and mixed with 2 L of 95% ethanol (v/v) before ultrasonic extraction. The solution was ultrasonicated twice for 30 min each time at 40 kHz at room temperature. After filtration, the ethanolic extract was dried by rotary evaporation. The dry product was redissolved in 500 mL of water, degreased with 500 mL of petroleum ether, extracted with 500 mL of ethyl acetate three times, and evaporated to dryness. The ethyl acetate extract was redissolved in 500 mL of ethyl acetate and adjusted to pH 9–10 using 1 mol/L NaOH. Then, the alkalized water layer was isolated and acidified to pH 2–3 with 1 mol/L HCl. The acidified water layer was extracted with ethyl acetate three times to obtain crude GLAA. The GLAA was dried and stored at 4 °C. Experimental research on *G. lucidum* complied with local and Chinese guidelines and legislation.

### Animals

Male specific-pathogen-free (SPF) Institute of Cancer Research (ICR) mice were purchased from Hangzhou Medical College and kept in SPF animal centre of Hangzhou Medical College under controlled light (12 h light–dark cycle), temperature (22–26 °C) and humidity (40–60%) conditions with free access to food and water. Mice that weighed 20–24 g (approximately 6 weeks) were randomly distributed into groups containing ten animals each (five mice per cage) using the standard = RAND() function in Microsoft Excel. The mice were allowed to acclimate to the laboratory conditions for 7 days before pharmacological tests. All experimental procedures were approved by the Animal Experimentation Ethics Committee of Hangzhou Medical College. The experiments were performed in accordance with the criteria of the “Guide for the Care and Use of Laboratory Animals” (NIH publication 86-23, revised 1985) and the ARRIVE guidelines (https://arriveguidelines.org).

### Pentobarbital-induced hypnosis test

The pentobarbital hypnosis test was performed as described previously^13^. GLAA was resolved in 0.05% CMC-Na. For the GLAA assay, forty mice were assigned to 4 groups and orally administered GLAA (25, 50 or 100 mg/kg) or 0.05% CMC-Na as a solvent control from 8:00 a.m. to 9:00 a.m. every day for 4 weeks. For the antibiotic depletion experiment, forty mice were assigned to 4 groups: pre-treated saline (control and 100 mg/kg groups) or antibiotics (control + AB and 100 mg/kg + AB groups) for 40 days. On the 13th to the 40th day, the 100 mg/kg and 100 mg/kg + AB groups were gavaged with 100 mg/kg GLAA, while the other two groups (control and control + AB) were administered 0.05% CMC-Na.

On the 28th day of the GLAA assay and the 40th day of the antibiotic depletion experiment, 1 h after the last administration of GLAA, 50 mg/kg pentobarbital sodium was injected subcutaneously. The animals were considered asleep if they stayed motionless when positioned on their back and when they lost their righting reflex. The time interval between injection of pentobarbital and start of sleep was considered sleep latency, while prolongation of sleep was described as sleeping time. Faecal samples were collected on the 28th day after administration of GLAA. Mice were sacrificed under carbon dioxide asphyxia 24 h after the pentobarbital-induced hypnosis test. Blood and tissue (brain, spleen, liver, kidney, thymus and colon) were collected. All samples were stored at − 80 °C until use.

### Antibiotic-induced depletion of the gut microbiota

Antibiotics treatment was performed according to Refs.^20,53^ and divided into two stages. In the first stage, mice were gavaged with amphotericin B (Solarbio Life Science, Beijing, China) at a dose of 1 mg/kg body weight every 12 h for three days. The second stage was from the 4th day to the 40th day, when the water was supplemented with 1 g/L ampicillin (Sangon Biotech, Shanghai, China). Furthermore, an antibiotic combination consisting of 50 mg/kg vancomycin (Sangon Biotech, Shanghai, China), 100 mg/kg neomycin (Sangon Biotech, Shanghai, China), 100 mg/kg metronidazole (Sangon Biotech, Shanghai, China), and 1 mg/kg amphotericin B was administered by gavage every 12 h in the second stage.

### Neurotransmitter and bacterial component detection

Detection of the neurotransmitters 5-hydroxytryptamine (5-HT), γ-aminobutyric acid (GABA), norepinephrine and dopamine (Jianchen, Nanjing, China) in the hypothalamus, as well as detection of LPS (Jianchen, Nanjing, China) and PG (AMEKO, Shanghai, China) in serum, was carried out with ELISA kits according to the manufacturer’s instructions.

### Gut microbiota analysis

Faecal microbial DNA was extracted using the QIAamp DNA Stool MiniKit (Qiagen, Hilden, Germany). The PCR primers 341F (5′-CCTACGGGNGGCWGCAG-3′) and 805R (5′-GACTACHVGGGTATCTAATCC-3′) targeting the V3–V4 region of the 16S rDNA gene with specific barcodes were used^54^. PCR was performed in a 25 μL PCR system containing 50 ng of template DNA, 25 pmol of each primer and 12.5 μL of Phusion Hot Start Flex 2X Master Mix (New England Biolabs, Ipswich, MA, USA) with initial denaturation at 98 °C for 30 s, 32 cycles of amplification (denaturation at 98 °C for 10 s; annealing at 54 °C for 30 s and extension at 72 °C for 45 s) and a final extension at 72 °C for 10 min. After purification, sequencing was performed using the Illumina Novaseq platform (Illumina, San Diego, CA, USA).

Raw sequence reads were assigned to each sample according to their unique barcode pairs. Overlapping paired-end reads were merged to form tags using FLASH (version 1.2.11)^55^. The following quality control criteria were used: (1) an exact match to at least one end of barcodes and primers; (2) no undetermined bases in the tags; and (3) no more than three mismatches in the overlap region. Operational taxonomic units (OTUs) were clustered with a 97% similarity cut-off using Usearch 11^56^. Alpha diversity was determined using the R packages “rich” and “diversity”. Principal coordinate analysis (PCoA), a standard multivariate statistical technique, was performed with the R package “ade4” to explain differences among microbial communities. The longest sequence in each OTU was chosen as the representative sequence for identification using the Ribosomal Database Project (RDP) database v.11.3^57^. The Chao1 index, Shannon index and principal coordinates analysis (PCoA) of unweighted UniFrac beta diversity were calculated by QIIME software (version 1.8.0) to investigate the richness and diversity of each gut microbiome^58^. Linear discriminant analysis effect size (LEfSe) analysis was performed on the Galaxy web platform^59^. The effect size of each differential taxon was estimated by the linear discriminant analysis (LDA) score, and the taxa with LDA scores greater than 3.5 were defined as discriminative taxa.

### Metabolic analysis

LC–MS/MS was used for metabolic analysis of 0.1 g of frozen faeces. A mixture containing 0.1 g of frozen faeces, 300 µL of ice-cold water and 3–4 cold 3 mm steel balls (Jingxin, Shanghai, China) was whirled for 5 min. Then, 500 µL of methanol containing 1 ppm dl-2-chlorophenylalanine was added into the mixture without steel balls, and the mixture was further centrifuged at 12,000 rpm and 4 °C for 10 min. Then, 600 µL of supernatant was sucked into another centrifuge tube and concentrated. Then, 100 µL of 5% methanol water was added to the dried product, mixed and centrifuged at 12,000 rpm at 4 °C for 10 min. Finally, the supernatant was collected for LC–MS/MS (Agilent Technologies Inc., Palo Alto, CA, USA) analysis.

The analytical conditions were as follows: UPLC: column, Waters ACQUITY UPLC HSS T3 C18 (1.8 µm, 2.1 mm × 100 mm); column temperature, 35 °C; flow rate, 0.3 mL/min; injection volume, 1 μL; solvent system, water (0.01% methanolic acid):acetonitrile; gradient program of positive ion, 95:5 V/V at 0 min, 79:21 V/V at 3.0 min, 50:50 V/V at 5.0 min, 30:70 V/V at 9.0 min, 5:95 V/V at 10.0 min, 95:5 V/V at 14.0 min; gradient program of negative ion, 95:5 V/V at 0 min, 79:21 V/V at 3.0 min, 50:50 V/V at 5.0 min, 30:70 V/V at 9.0 min, 5:95 V/V at 10.0 min, and 95:5 V/V at 14.0 min.

The original data file obtained by LC–MS analysis was first converted to mzML format by ProteoWizard software^60^. Peak extraction, alignment and retention time correction were performed by the XCMS program^61^. The "SVR" method was used to correct the peak area. Peaks with deletion rates > 50% were filtered from each group of samples. Then, metabolic identification information was obtained by searching the laboratory's in-house database and integrating the public database (HMDB and KEGG) and then combining it with metDNA^62^. Unbiased principal component analysis (PCA), orthogonal partial least squares discriminant analysis (OPLS-DA) and variable importance in projection (VIP) were calculated using SIMCA software (version 14.1) (Sartorius Stedim Biotech, Umeå, Sweden).

### Comparative transcriptomic analysis

Total RNA from the hypothalamus was extracted using TRIzol reagent (Invitrogen, Carlsbad, CA, USA) following the manufacturer’s protocol. The total RNA quantity and purity were analysed with a 2100 Bioanalyzer and the RNA 6000 Nano LabChip Kit (Agilent, Palo Alto, CA, USA) with RIN number > 7.0. Poly(A) RNA was purified from total RNA (5 µg) using poly-T oligo-attached magnetic beads using two rounds of purification. Following purification, the mRNA was fragmented into small pieces using divalent cations under elevated temperature. Then, the cleaved RNA fragments were reverse-transcribed to create the final cDNA library in accordance with the protocol for the mRNA-Seq sample preparation kit (Illumina, San Diego, CA, USA); the average insert size for the paired-end libraries was 300 bp (± 50 bp). Next, we performed paired-end sequencing on an Illumina HiSeq 4000 (Illumina, San Diego, CA, USA) following the vendor’s recommended protocol. Then, the sample reads were aligned to the UCSC (http://genome.ucsc.edu/) mouse reference genome using the HISAT package, which initially removes a portion of the reads based on the quality information accompanying each read and then maps the reads to the reference genome. The mapped reads were assembled using StringTie^63^. Then, all transcriptomes from samples were merged to reconstruct a comprehensive transcriptome using Perl scripts. After the final transcriptome was generated, StringTie and edgeR were used to estimate the expression levels of all transcripts. StringTie was used to determine the expression level of mRNAs by calculating fragments per kilobase of exon model per million mapped reads (FPKM) values^64^. The differentially expressed mRNAs and genes were selected with log_2_ (fold change) > 1 or log_2_ (fold change) <  − 1 and with statistical significance (*P* value < 0.05) by the R package.

### Statistical analysis

The obtained data were analysed using SPSS 22.0 software (IBM Corporation, Armonk, NY, USA). The data were first tested for normal distribution using the Kolmogorov–Smirnov test. The Mann–Whitney U test (for nonnormal distributions) or Student’s t test (for normal distributions) was performed to test for differences between two groups. Spearman’s rank correlation analysis was conducted using the corrplot package of R. The images are generated by GraphPad Prism 8 (GraphPad Software, La Jolla, CA, USA), R and SIMCA software.

## Supplementary Information

Supplementary Information.

Supplementary Figures.

## Data Availability

All data generated or analysed during this study are included in this published article and its additional information files. The data sets generated during the current study are available in the GenBank Sequence Read Archive Repository under accession number PRJNA731631.

## References

[1] Bruce ES, Lunt L, McDonagh JE (2017). Sleep in adolescents and young adults. Clin. Med. (Lond.).

[2] Pavlova MK, Latreille V (2019). Sleep disorders. Am. J. Med..

[3] Troynikov O, Watson CG, Nawaz N (2018). Sleep environments and sleep physiology: A review. J. Therm. Biol..

[4] Akram M, Daniyal M, Munir N, Mohiuddin E, Sultana S (2019). Medicinal plants combating against insomnia: A green anti-insomnia approach. J. Nerv. Ment. Dis..

[5] Javaheri S, Redline S (2017). Insomnia and risk of cardiovascular disease. Chest.

[6] St-Onge MP (2017). Sleep–obesity relation: Underlying mechanisms and consequences for treatment. Obes. Rev..

[7] Naseer MI (2014). Role of gut microbiota in obesity, type 2 diabetes and Alzheimer's disease. CNS Neurol. Disord. Drug Targets.

[8] Parthasarathy S (2015). Persistent insomnia is associated with mortality risk. Am. J. Med..

[9] Bishop KS (2015). From 2000 years of *Ganoderma lucidum* to recent developments in nutraceuticals. Phytochemistry.

[10] Chinese Pharmacopoeia Commission. Pharmacopoeia of the People’s Republic of China. *The Medicine Science and Technology Press of China*. ISBN: 978-977-5214-1599-5215 (2020).

[11] Tang W (2005). A randomized, double-blind and placebo-controlled study of a *Ganoderma lucidum* polysaccharide extract in neurasthenia. J. Med. Food.

[12] Wang XL, Wang CP (2001). Clinical trials of *Ganoderma lucidum* on 60 patients suffered insomnia. Zhong Guo Yi Yao Xue Bao.

[13] Chu QP (2007). Extract of *Ganoderma lucidum* potentiates pentobarbital-induced sleep via a GABAergic mechanism. Pharmacol. Biochem. Behav..

[14] Galland L (2014). The gut microbiome and the brain. J. Med. Food.

[15] Anderson JR (2017). A preliminary examination of gut microbiota, sleep, and cognitive flexibility in healthy older adults. Sleep Med..

[16] Poroyko VA (2016). Chronic sleep disruption alters gut microbiota, induces systemic and adipose tissue inflammation and insulin resistance in mice. Sci. Rep..

[17] Novikov A, Breton A, Caroff M (2017). Micromethods for isolation and structural characterization of lipid A, and polysaccharide regions of bacterial lipopolysaccharides. Methods Mol. Biol..

[18] Ginsberg C, Brown S, Walker S (2008). Bacterial Cell Wall Components.

[19] Liang L (2020). Metabolic dynamics and prediction of gestational age and time to delivery in pregnant women. Cell.

[20] Reikvam DH (2011). Depletion of murine intestinal microbiota: Effects on gut mucosa and epithelial gene expression. PLoS One.

[21] Van Cauter E, Leproult R, Plat L (2000). Age-related changes in slow wave sleep and REM sleep and relationship with growth hormone and cortisol levels in healthy men. JAMA.

[22] Machado RB, Rocha MR, Suchecki D (2017). Brain prolactin is involved in stress-induced REM sleep rebound. Horm. Behav..

[23] Kanehisa M, Goto S (2000). KEGG: Kyoto encyclopedia of genes and genomes. Nucleic Acids Res..

[24] Kanehisa M (2019). Toward understanding the origin and evolution of cellular organisms. Protein Sci..

[25] Kawai M, Rosen CJ (2010). Minireview: A skeleton in serotonin's closet?. Endocrinology.

[26] Cui XY (2012). Extract of *Ganoderma lucidum* prolongs sleep time in rats. J. Ethnopharmacol..

[27] Han J, Ning N (2014). Research advances of the chemical constituents and pharmacological effects of *Ganoderma lucidum*. Guangzhou Chem. Ind..

[28] Wang J, Cao B, Zhao H, Feng J (2017). Emerging roles of *Ganoderma lucidum* in anti-aging. Aging Dis..

[29] Feng H, Yang M, Yang X, Yang Q (2013). Determination of total triterpenoid acids in different part and extract of *Ganoderma lucidum*. J. Shanghai Norm. Univ. (Nat. Sci.).

[30] Wang X, Yang H, Liu G (2016). Enhanced triterpene acid production by *Ganoderma lucidum* using a feeding stimulus integrated with a two-stage pH-control strategy. J. Chem. Technol. Biotechnol..

[31] Saltarelli R (2019). Phytochemical composition, antioxidant and antiproliferative activities and effects on nuclear DNA of ethanolic extract from an Italian mycelial isolate of *Ganoderma lucidum*. J. Ethnopharmacol..

[32] Lin A (2019). Hypnotic effects of *Lactobacillus fermentum* PS150(TM) on pentobarbital-induced sleep in mice. Nutrients.

[33] Ko GY, Shi L, Ko ML (2009). Circadian regulation of ion channels and their functions. J. Neurochem..

[34] Liu Z (2012). Safranal enhances non-rapid eye movement sleep in pentobarbital-treated mice. CNS Neurosci. Ther..

[35] Zhao X, Cui XY, Wang LE, Zhang YH (2009). Potentiating effect of diltiazem on pentobarbital-induced hypnosis is augmented by serotonergic system: The TMN and VLPO as key elements in the pathway. Neuropharmacology.

[36] Leonard CS, Rao SR, Inoue T (2000). Serotonergic inhibition of action potential evoked calcium transients in NOS-containing mesopontine cholinergic neurons. J. Neurophysiol..

[37] Bayliss DA, Li YW, Talley EM (1997). Effects of serotonin on caudal raphe neurons: Inhibition of N- and P/Q-type calcium channels and the afterhyperpolarization. J. Neurophysiol..

[38] Krueger JM, Opp MR (2016). Sleep and microbes. Int. Rev. Neurobiol..

[39] Chang CJ (2015). *Ganoderma lucidum* reduces obesity in mice by modulating the composition of the gut microbiota. Nat. Commun..

[40] Cao YN (2018). *Lactobacillus acidophilus* and *Bifidobacterium longum* supernatants upregulate the serotonin transporter expression in intestinal epithelial cells. Saudi J. Gastroenterol..

[41] Bubier JA (2020). A microbe associated with sleep revealed by a novel systems genetic analysis of the microbiome in collaborative cross mice. Genetics.

[42] Papandreou C (2019). Circulating metabolites associated with objectively measured sleep duration and sleep variability in overweight/obese participants: A metabolomics approach within the SATIN study. Sleep.

[43] Teitelbaum JE, Johnson C, St Cyr J (2006). The use of D-ribose in chronic fatigue syndrome and fibromyalgia: A pilot study. J. Altern. Complement Med..

[44] Liguori C (2019). Cerebral glucose metabolism in idiopathic REM sleep behavior disorder is different from tau-related and alpha-synuclein-related neurodegenerative disorders: A brain [18F]FDG PET study. Parkinsonism Relat. Disord..

[45] Myint KZ (2020). Structural dependence of antidiabetic effect of steviol glycosides and their metabolites on streptozotocin-induced diabetic mice. J. Sci. Food Agric..

[46] Bottcher C (2014). The biosynthetic pathway of indole-3-carbaldehyde and indole-3-carboxylic acid derivatives in arabidopsis. Plant Physiol..

[47] Takakuwa Y, Kanazawa T (1984). Acetylphosphate-induced Ca2+-Ca2+ exchange that is mediated by (Ca2+, Mg2+)-ATPase in sarcoplasmic reticulum vesicles. J. Biochem..

[48] Ahmed T (2017). Daidzein and its effects on brain. Curr. Med. Chem..

[49] Knight LS, Piibe Q, Lambie I, Perkins C, Yancey PH (2017). Betaine in the brain: Characterization of betaine uptake, its influence on other osmolytes and its potential role in neuroprotection from osmotic stress. Neurochem. Res..

[50] Mayer EA, Tillisch K, Gupta A (2015). Gut/brain axis and the microbiota. J. Clin. Investig..

[51] Cryan JF (2019). The microbiota–gut–brain axis. Physiol. Rev..

[52] Li P (2020). Anti-cancer effects of a neutral triterpene fraction from *Ganoderma lucidum* and its active constituents on SW620 human colorectal cancer cells. Anticancer Agents Med. Chem..

[53] Pircalabioru G (2016). Defensive mutualism rescues NADPH oxidase inactivation in gut infection. Cell Host Microbe.

[54] Logue JB (2016). Experimental insights into the importance of aquatic bacterial community composition to the degradation of dissolved organic matter. ISME J..

[55] Magoc T, Salzberg SL (2011). FLASH: Fast length adjustment of short reads to improve genome assemblies. Bioinformatics.

[56] Edgar R (2018). Taxonomy annotation and guide tree errors in 16S rRNA databases. PeerJ.

[57] Cole JR (2014). Ribosomal Database Project: Data and tools for high throughput rRNA analysis. Nucleic Acids Res..

[58] Caporaso JG (2010). QIIME allows analysis of high-throughput community sequencing data. Nat. Methods.

[59] Afgan E (2018). The Galaxy platform for accessible, reproducible and collaborative biomedical analyses: 2018 update. Nucleic Acids Res..

[60] Chambers MC (2012). A cross-platform toolkit for mass spectrometry and proteomics. Nat. Biotechnol..

[61] Forsberg EM (2018). Data processing, multi-omic pathway mapping, and metabolite activity analysis using XCMS Online. Nat. Protoc..

[62] Shen X (2019). Metabolic reaction network-based recursive metabolite annotation for untargeted metabolomics. Nat. Commun..

[63] Kovaka S (2019). Transcriptome assembly from long-read RNA-seq alignments with StringTie2. Genome Biol..

[64] Pertea M (2015). StringTie enables improved reconstruction of a transcriptome from RNA-seq reads. Nat. Biotechnol..

