# Supplementary material for: Ganoderma lucidum promotes sleep through a gut microbiota-dependent and serotonin-involved pathway in mice
Source: Sci Rep. 2021 Jul 1;11:13660. doi: 10.1038/s41598-021-92913-6 (PMC8249598; doi:10.1038/s41598-021-92913-6)
Supplement: Supplementary file 2 — Supplementary Figures. [file 41598_2021_92913_MOESM2_ESM.docx]

*Ganoderma lucidum* promotes sleep through a gut microbiota-dependent and serotonin-involved pathway in mice

**Order of authors:**

Chunyan Yao^1†^, Zhiyuan Wang^2†^, Huiyong Jiang^3†^, Ren Yan^3†^, Qianfei Huang^1^，Yin Wang^1^, Hui Xie^2^, Ying Zou^4^, Ying Yu^1^*, Longxian Lv^3^*

**Authors’ affiliations:**

^1^ Key Laboratory of Nutrition of Zhejiang Province, Institute of Health Food, Hangzhou Medical College, Hangzhou 310013, China

^2^ Animal Center, Hangzhou Medical College, Hangzhou 310013, China

^3^ State Key Laboratory for Diagnosis and Treatment of Infectious Diseases, Collaborative Innovation Center for Diagnosis and Treatment of Infectious Diseases, The First Affiliated Hospital, College of Medicine, Zhejiang University, Hangzhou 310003, China

^4^ The Second Affiliated Hospital of Zhejiang Chinese Medical University, Hangzhou 310013, China

^†^ These author contribute equal work to our study.

*** Corresponding author:**

Ying Yu

Institute of Health Food, Hangzhou Medical College

182 Tianmushan Road, Hangzhou 310013, China

Tel：86-571-88215480

E-mail: yuying@hmc.edu.cn

Longxian Lv

State Key Laboratory for Diagnosis and Treatment of Infectious Diseases, Collaborative Innovation Center for Diagnosis and Treatment of Infectious Diseases, The First Affiliated Hospital, College of Medicine, Zhejiang University, Hangzhou 310003, China

Tel：86-571-88236423

E-mail: lvlongxian@zju.edu.cn

Supplementary figure. S1


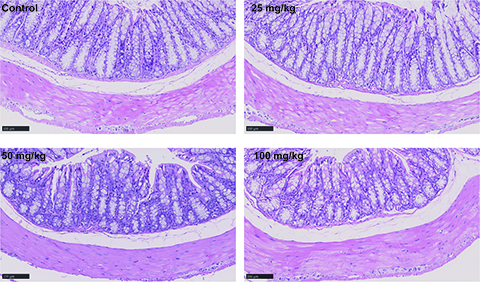


Supplementary figure S1. Representative images of colic HE staining; scale bar is 100 μm.

Supplementary figure. S2


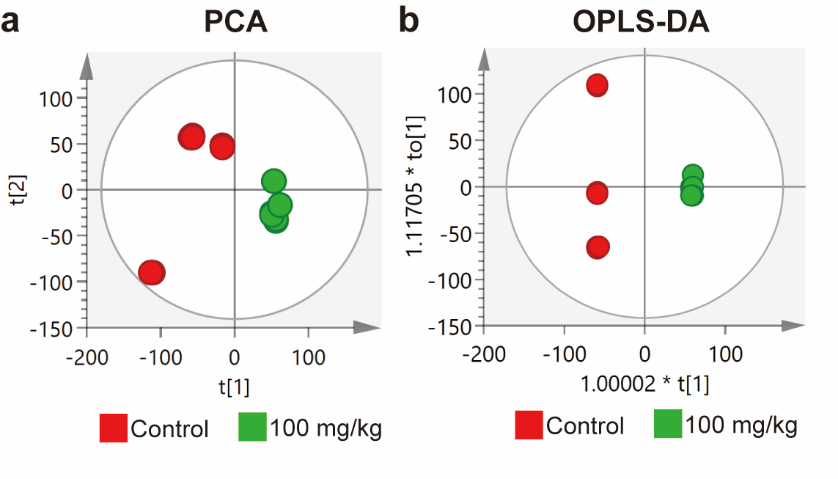


Supplementary figure. S2. Principal component analysis (PCA, a) and the [orthogonal partial least squares discriminant analysis](https://xueshu.baidu.com/usercenter/paper/show?paperid=406beac8af1f00c4f60d6480f4a3fb9b) (OPLS-DA, b) score plots of original metabolome profiles between the control group and the 100 mg/kg GLAA group.

Supplementary figure. S3


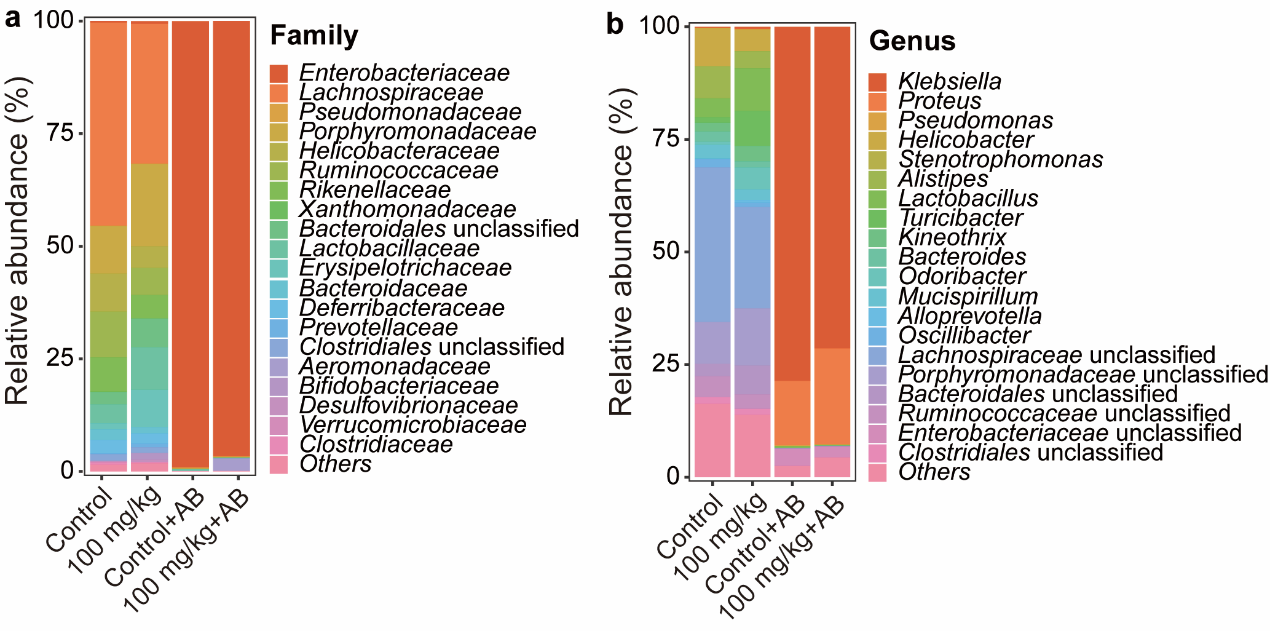


Supplementary figure S3. Composition of the faecal microbiota after antibiotics treatment at the family level (a) and the genus level (b).
